# Iterative optimization techniques using man–machine interaction for university timetabling problems

**DOI:** 10.1186/s40064-015-1018-3

**Published:** 2015-06-12

**Authors:** Syunsuke Shimazaki, Kazutoshi Sakakibara, Takuya Matsumoto

**Affiliations:** Toyama Prefectural University, 5180 Kurokawa, 939-0398 Imizu, Japan; Kobe University, Rokkodai, 657-8501 Kobe, Japan

**Keywords:** University course timetabling problem, Integer programming, Man–machine interaction

## Abstract

We focus on a timetabling problem of university makeup classes and construct a scheduling system based on man–machine interaction which enables to reveal the essential and additional information of the problem domain. In this problem, makeup classes which are requested by the lecturers have to be scheduled to a specified time slot under the hard/soft constraints, e.g., schedules of the lecturers and the students. A constraint based scheduling model is newly introduced and several parameters of the model are settled through the repetition of the solution evaluation by the operators. Through the numerical experiment with the actual data, the potential of the proposed approach is examined.

## Background

Recently, many researches on timetabling problems have been reported from the viewpoint of optimization (Burke and Petrovic 2002; Daskalaki et al.^ 2004^; Causmaecker et al. 2009; Burke et al. 2010; Abdullaha and Turabieh 2012). Most of them take the approach of utilizing a computer and generating a feasible timetable based on a mathematical programming model (Nemhauser et al. 1989) which is formulated after the interview with the experts of the problem domain. On the other hand, generated timetables become rarely operative in the realistic situation, and human modifications are added to them in many cases. This always results from disability of mathematical programming model. In contrast, we consider an approach to generate a solution (i.e., a timetable) of the target problem by referring to the implicit knowledge from an operator. It is important to incorporate man–machine interaction explicitly into an optimization process in this approach.

## Introduction

We propose iterative optimization method based on man–machine interaction for makeup classes timetabling problems specially in Toyama Prefectural University. In this university, there is an arranged period for the makeup classes in each semester, and it takes 5–6 days. A timetable has to be scheduled under the constraints of inhibition of overlapped time slots both for the lecturer and the students. In the past of the university, one operator has scheduled a timetable of the makeup class, and the total scheduling time has been about 3 weeks. Our proposed system was applied to the makeup classes of the second semester of 2013, the first and the second semester of 2014. Its effectiveness was evaluated quantitatively by comparing with the conventional hand working method and qualitatively by the hearing survey to the staffs.

## Mathematical programming models and makeup class timetabling problem

We first formulate a makeup class timetabling problem into an integer programming model. A makeup class is a class which is offered as a substitute for the canceled class in the ordinary course in the university. In Toyama Prefectural University, 5–6 days are arranged for a set of makeup classes requested by the lecturers in each semester, and timetables of the makeup classes are scheduled by a staff of the educational affairs section. A classroom for each makeup class is also scheduled simultaneously with the timetabling. When feasible timetables are not able to be found, a set of classes for operating in the arranged period needs to be selected. The rest of classes are operated in the ordinary course period.

### Parameters

Parameters of the integer programming model are summarised as follows:

- Time slot ${\rm {S}}_i$ ($i\in \mathcal{I}$): Each ${\rm {S}}_{i}$ corresponds to 90 min. Dummy time slots are also introduced for the sake of convenience in order to represent the beginning/end of the arranged period and the turn of a day.
  - $\mathcal{I}^\mathrm{D}$: a set of dummy time slots,
  - $\mathcal{I}^\mathrm{E}$: a set of indices of the time slots which correspond to 11th and 12th period of a day. These time slots correspond to the evening time, and it is desirable to avoid these slots for allocation of the classes.
- Classroom $${\rm {R}}_j$$ ($$j\in \mathcal{J}$$):
  - $$p_j$$: room capacity.
- Class $${\rm {C}}_k$$ ($$k\in \mathcal{K}$$)
  - $\mathcal{S}_k (\subseteq \mathcal{I})$: a set of the desired time slots,
  - $\mathcal{R}_k (\subseteq \mathcal{J})$: a set of classrooms,
  - $a_k$: number of time slots to be operated in succession,
  - $m_k$: number of participation in ${\rm {C}}_{k}$,
  - $b^\mathrm{S}_{ik}\in \{0,1,...\},i\in \mathcal{S}_k$: degree of favor for ${\rm S}_{i}$,
  - $b^\mathrm{R}_{jk}\in \{0,1,...\},j\in \mathcal{R}_k$: degree of favor for ${\rm R}_{j}$,
  - $b^\mathrm{C}_k$: priority of operation of ${\rm C}_{k}$.
- Student $${\rm U}_{\ell }$$ ($$l\in \mathcal{L}$$):
  - $\mathcal{C}^\mathrm{S}_{\ell } (\subset \mathcal{K})$: a set of registered classes.
- Lecturer $${\rm L}_m$$ ($$m\in \mathcal{M}$$):
  - $\mathcal{C}^\mathrm{L}_{m} (\subset \mathcal{K})$: a set of operable classrooms,
  - $\mathcal{M}^\mathrm{Q}_{m}$: a set of the classes which are desirable to be placed closely on a timetable,
  - $\mathcal{M}^\mathrm{S}_{m}$: a set of the classes which are desirable to be placed separately on a timetable.

Here, the values of $b^\mathrm{S}_{ik}$, $b^\mathrm{R}_{jk}$ and $b^\mathrm{C}_{k}$ are settled by the interview with the lecturers.

### Decision variables

Decision variables and dependent variables on them are defined as follows:

- Allocation of classroom ${\rm R}_j$ and time slot ${\rm S}_i$ to class ${\rm C}_k$:
  $$\begin{aligned} x_{ijk}= \left\{ \!\! \begin{array}{ll} 1\!\!: & \, \text{ C }_{k} \text{ is } \text{ operated } \text{ on } \text{ S }_{i} \text{ in } \text{ R }_{j}, \\ 0\!\!: & \, \text{ otherwise. } \end{array}\right. \end{aligned}$$
- Selection of the classes:
  $$\begin{aligned} y_k= \left\{\begin{array}{ll} 1\!\!: \, \text{ C }_k \text{ is } \text{ operated } \text{ in } \text{ the } \text{ period } \text{ for } \text{ makeup } \text{ classes, } \\ 0\!\!: \, \text{ otherwise. } \end{array}\right. \end{aligned}$$
- $s_{k}$: the center of time slots of ${\rm C}_{k}$.
- $d^\mathrm{Q}_{kk'}$, $d^\mathrm{S}_{kk'}$: difference of the time slots between ${\rm C}_{k}$ and ${\rm C}_{k'}$.

### Constraints

In making a timetable, following constraints, where hard constraints and soft constraints are denoted with the symbols ‘$\triangleright$’ and ‘$\star$’ respectively, should be taken into account. Here, every timetable is necessary to satisfy the hard constraints, whereas it need not always satisfy the soft constraints. We first give the hard constraints:

- $\triangleright$ Each class is assigned to $a_{k}$ time slots:
  1 $$\begin{aligned} \sum _{i\in \mathcal{S}_{k}}\sum _{j\in \mathcal{R}_{k}} x_{ijk} = a_{k}y_{k}, \quad k\in \mathcal{K} \end{aligned}$$
- $$\triangleright$$$${\rm C}_{k}$$ is operated in a succession of $$a_{k}$$: time slots
  2 $$\begin{aligned} x_{ijk} - x_{i-1,jk} - x_{i+1,jk} \le 0, \quad i\in \mathcal{I}, \,j\in \mathcal{J}, \,k|_{a_{k}\ge 2}\in \mathcal{K} \end{aligned}$$
  3 $$\begin{aligned} x_{ijk} + x_{i+1,jk} - x_{i-1,jk} - x_{i+2,jk} \le 1, \quad i\in \mathcal{I}, j\,\in \mathcal{J}, \, k|_{a_{k}\ge 4}\in \mathcal{K} \end{aligned}$$
  These inequalities are able to express the constraints with $a_{k}\le 5$.
- $\triangleright$ The constraints on a dummy time slot:
  4 $$\begin{aligned} x_{ijk} = 0, \quad i\in \mathcal{I}^\mathrm{D}, \quad j\in J,\, k\in \mathcal{K} \end{aligned}$$
  Every class cannot be assigned on each dummy time slot.
- $\triangleright$ A lecturer does not operate more than two classes at the same time slot.
  5 $$\begin{aligned} \sum _{j\in \mathcal{J}}\sum _{k\in \mathcal{C}^\mathrm{L}_m} x_{ijk} \le 1, \quad i\in \mathcal{I},\, m\in \mathcal{M} \end{aligned}$$
- $\triangleright$ A lecturer does not operate more than two classes at the same classroom:
  6 $$\begin{aligned} \sum _{k\in \mathcal{K}} x_{ijk} \le 1, \quad i\in \mathcal{I}, \, j\in \mathcal{J} \end{aligned}$$
- $\triangleright$ Capacity of a classroom:
  7 $$\begin{aligned} \sum _{k\in \mathcal{K}} m_{k}x_{ijk} \le p_{j}, \quad i\in \mathcal{I},\,j\in \mathcal{J} \end{aligned}$$

Next, we denotes the soft constraints as follows:

- $\star$ A class is assigned to a time slot according to the degree of favor for it:
  8 $$\begin{aligned} f^\mathrm{A} = \sum _{k\in \mathcal{K}}\sum _{i\in \mathcal{S}_k}\sum _{j\in \mathcal{R}_k} b^\mathrm{S}_{ik}x_{ijk} \rightarrow \text{ maximize } \end{aligned}$$
- $\star$ A student does not attend more than two classes at the same time slot:
  9 $$\begin{aligned} \lambda _{i\ell } = \sum _{j\in \mathcal{J}}\sum _{k\in \mathcal{C}^\mathrm{S}_\ell } x_{ijk} - 1, \quad i\in \mathcal{I},~ \ell \in \mathcal{L} \end{aligned}$$
  10 $$\begin{aligned} f^\mathrm{B} = \sum _{i\in \mathcal{I}}\sum _{l\in \mathcal{L}} \lambda _{i\ell } \rightarrow \text{ minimize } \end{aligned}$$
  These constraints are originally classified as hard constraints. However, it is difficult to find the feasible schedule satisfying of them in the case of Toyama Prefectural University. We therefore relax it and add a penalty term to the original objective functions.
- $\star$ Evaluation of degree of adjacency of the classes among $\mathcal{M}^\mathrm{Q}_{m}$:
  11 $$\begin{aligned} s_{k} = \frac{1}{a_{k}} \sum _{i\in \mathcal{S}_{k}} \sum _{j\in \mathcal{J}} i\cdot x_{ijk}, \quad k\in \mathcal{K} \end{aligned}$$
  12 $$\begin{aligned} d^\mathrm{Q}_{k k'} \ge s_{k} - s_{k'}, k<k', ~ k,k' \in \mathcal{M}^\mathrm{Q}_{m}, \quad m \in \mathcal{M} \end{aligned}$$
  13 $$\begin{aligned} d^\mathrm{Q}_{k k'} \ge s_{k'} - s_{k}, k<k', ~ k,k' \in \mathcal{M}^\mathrm{Q}_{m}, \quad m \in \mathcal{M} \end{aligned}$$
  14 $$\begin{aligned} f^\mathrm{C} = \sum _{m\in \mathcal{M}} \sum _{k\in \mathcal{M}^\mathrm{Q}_{m}} \sum _{\begin{array}{c} k'\in \mathcal{M}^\mathrm{Q}_{m}, \\ {k'>k} \end{array}} d^\mathrm{Q}_{k k'} \rightarrow \text{ minimize } \end{aligned}$$
- $\star$ Evaluation of degree of disjunctiveness of the time slots of the classes among $\mathcal{M}^\mathrm{S}_{m}$:
  15 $$\begin{aligned} d^\mathrm{S}_{k k'} = s_{k'} - s_{k}, k<k', ~ k,k' \in \mathcal{M}^\mathrm{S}_{m}, \quad m \in \mathcal{M} \end{aligned}$$
  16 $$\begin{aligned} f^\mathrm{D} = \sum _{m\in \mathcal{M}} \sum _{k\in \mathcal{M}^\mathrm{S}_{m}} \sum _{\begin{array}{c} k'\in \mathcal{M}^\mathrm{S}_{m}, \\ {k'>k} \end{array}} d^\mathrm{S}_{k k'} \rightarrow \text{ maximize } \end{aligned}$$
- $\star$ Evaluation of degree of avoidance of the slots in $\mathcal{I}^\mathrm{E}$:
  17 $$\begin{aligned} f^\mathrm{E} = \sum _{i\in \mathcal{I}^\mathrm{E}} \sum _{j\in \mathcal{J}} \sum _{k\in \mathcal{K}} x_{ijk} \rightarrow \text{ minimize } \end{aligned}$$
- $\star$ Evaluation of degree of the priority of the class:
  18 $$\begin{aligned} f^\mathrm{F} = \sum _{k\in \mathcal{K}} b^\mathrm{C}_{k}y_{k} \rightarrow \text{ maximize } \end{aligned}$$

Finally, we show the implicit constraints which cannot be represented in a linear inequality form as follows:

- $\star$ Vacancy slots between the pair of the classes are not desirable for each student.
- $\star$ Constraint (10) is able to satisfied for the student by attending the class which is offered to the different department in the same faculty and also has the same course content. This movement can be reflected on the integer programming model by changing the value of $C^\mathrm{S}_{\ell }$.

Equations (8), (10), (14), (16) and (18) have to be optimized (i.e., minimized or maximized) concurrently in order to achieve the timetable satisfying the all soft constraints. Therefore, the makeup class timetabling problem becomes a multiobjective optimization problem (Koksalan et al. 2011).

## Iterative optimization method based on man–machine interaction

In this section, we model a procedure of interaction between an expert and a computer in making a timetable.

### The original problem and its mathematical programming model

In our research, we focus on a problem, which cannot be formulated by existing mathematical programming models completely. Every optimization problem is originally modeled into mathematical formulas, graph expression or computer languages by describing decision variables, parameters (i.e., constant values), constraints on the decision variables and objective functions.

**Figure 1:**
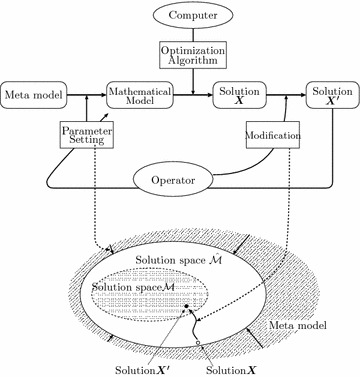
Solution process for timetabling.

**Figure 2:**
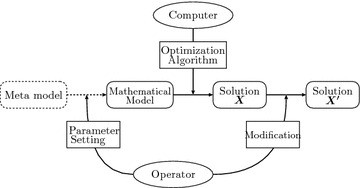
Solution process for timetabling (conventional).

In this paper, we give the following assumption in order to restrict our discussion on the university timetabling problem:

- the decision variables and the parameters can be described in a mathematical programming manner completely.

Under this assumption, there exists the elements which cannot be formalized into a mathematical programming model among the constraints and the objective functions. Here, such constraints can be classified into the followings:

(A) The constraints which are described into mathematical formulas, and at the same time need not always be satisfied in making a timetable, and
(B) The constraints which cannot be formalized primarily. We call them implicit constraints.

As for the constraints of (A) (i.e., the soft constraints), it is a general approach to relax these constraints and to incorporate them into the objective functions where the degree of a violation of them is quantified and is minimized as shown in "Constraints". However, this problem deformation is equivalent to the multi-objectivization of the original problem, and it poses a problem of balancing multiple objective functions.

A solution space which is represented by the formulated constraints includes the original solution space with implicit constraints in both case of (A) and (B).

### Optimization process

We propose an optimization approach by newly introducing *meta model* for the problem described in "The original problem and its mathematical programming model". Meta-model is a model which is allowed to include both the implicit constraints and the implicit objective functions. The implicit objective functions include the parameters which are difficult to be quantified. A mathematical programming model is generated by setting/modifying these parameters by the operators.

On the other hand, the implicit constraints are reflected in the modifying process of the solutions generated by optimizing the acquired mathematical programming model. In other words, a computer processes the mathematical programming model, and an operator sets the values of the parameters of the meta-model and modifies the solution generated from the computer.

Figure 1 shows the proposed optimization process. In this figure, the following three steps are iterated:

1. Operators generate a mathematical programming model by setting the values of the parameters based on the reference of the solution $\varvec{X}'$ acquired in the previous step in order to reflect the implicit objective functions.
2. A computer performs the optimization process and output a timetable $\varvec{X}$.
3. Operators directly modify the timetable $\varvec{X}$ in order to reflect the implicit constraints.

These procedure can be illustrated in the solution space of Figure 1. In this figure, step 1° corresponds to generating a solution space $\hat{\mathcal{M}}$ which is drawn with a solid line. Step 2° outputs a solution $\varvec{X}$ from a solution space $\hat{\mathcal{M}}$. $\varvec{X}$ satisfies all hard constraints of Eqs. (1)–(7) and all soft constrains of Eqs. (8)–(18) under the parameters settled by the operators. Step 3° outputs a solution $\varvec{X}'$ included in a solution space which corresponds to an original problem is shown with a broken line. $\varvec{X}$ satisfies all hard constraints of Eqs. (1)–(7) and all soft constrains of Eq. (8). Meanwhile a conventional optimization process by utilizing a computer can be illustrated in Figure 2. In this figure, there does not exist a meta-model explicitly, and exists one way process from a mathematical model to a modified solution $\varvec{X}'$. It indicates that the optimization process possesses no feed back loops. This lack of feed back loops is thought to bring an obsolescence of the optimization system associated with changes in environment. On the other hand, the proposed method has the feedback loops explicitly, and the values of the model parameters are set/modified by the operators based on the current solutions generated by a mathematical programming solver. These values are listed in a CSV format, and the operator can edit them in a spreadsheet application in our experiments described in "Experiments". In this paper, an integer programming model is used for the meta-model, and $b^\mathrm{S}_{ik}$, $b^\mathrm{R}_{jk}$, $b^\mathrm{C}_{k}$ correspond to the parameters in the implicit objective functions. These values are modified iteratively in the optimization loop after the interview with the lecturer.

An objective function defined as the weighted sum of the multiple objective functions ($f^\mathrm{A},\,\ldots ,\,f^\mathrm{F}$ described in "Constraints") which correspond to the soft constraints is newly introduced when we optimize the makeup class timetabling problem by using mathematical programming techniques such as branch-and-bound methods. The new objective function can be described as follows:

19 $$\begin{aligned} w^\mathrm{A} f^\mathrm{A} + w^\mathrm{B} f^\mathrm{B} + w^\mathrm{C} f^\mathrm{C} + w^\mathrm{D} f^\mathrm{D} + w^\mathrm{E} f^\mathrm{E} + w^\mathrm{F} f^\mathrm{F} \rightarrow \mathrm{minimize} \end{aligned}$$

where $w^\mathrm{A},\,w^\mathrm{D},\,w^\mathrm{F}<0$ and $w^\mathrm{B},\,w^\mathrm{C},\,w^\mathrm{E}>0$. The weight parameters $w^\mathrm{A}\,\ldots ,\,w^\mathrm{F}$ are also set and modified in the course of the iterative optimization process in Figure 1.

## Experiments

In this section, we show the numerical experiments with the actual data to examine the performance of the proposed method. We used the timetabling data of Toyama Prefectural University from the second semester of 2013 to the second semester of 2014. Here, the decision of the classroom selection were excluded in this experiments in order to reduce the model size described in "Mathematical programming models and makeup class timetabling problem". One staff of the educational affairs section in Toyama Prefectural University scheduled a timetable by using our proposed method.

### Problem specification

The problem specification we used is summarized as follows:

- The second semester of 2013 (we call SS2013)
  - Number of students: 994,
  - Number of slots arranged for makeup classes: 34,
  - Number of classes requested by the lecturers: 257.

- The first semester of 2014 (FS2014)
  - Number of students: 997,
  - Number of slots arranged for makeup classes: 36,
  - Number of classes requested by the lecturers: 280.

- The second semester of 2014 (SS2014)
  - Number of students: 997,
  - Number of slots arranged for makeup classes: 34,
  - Number of classes requested by the lecturers: 257.

**Figure 3:**
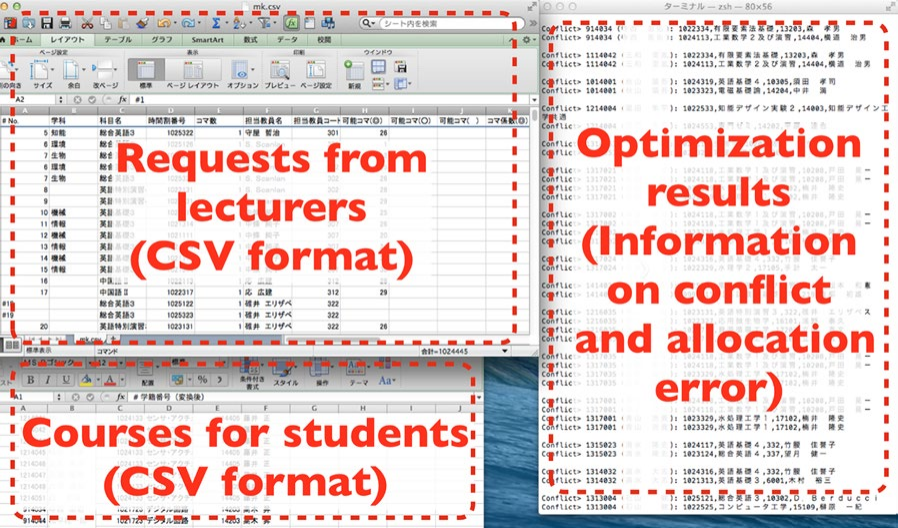
A screen shot of the implemented system.

We used IBM ILOG CPLEX12 (IBM Corp 2011) on a Core i5 (1.8GHz) computer for solving the integer programming model described in "Mathematical programming models and makeup class timetabling problem". The branch-and-bound techniques are employed in it. The scheduling staff was instructed that it is allowed to terminate the optimization procedure by the computer after a dual gap, which is the difference between upper and lower bounds of the objective values, is less than 1%. The values of the upper and lower bounds can be found in the course of applying IBM ILOG CPLEX12. Figure 3 shows a screen shot of the implemented system.

### Results

The model specification and the scheduling results are summarized as follows:

- SS2013
  - Number of variables: 41,472
  - Number of constraints: 45,259
  - Number of classes not operated in the period for the makeup classes: 8
  - Computational time (for each optimization by a computer): 1,088–5,384 s
  - Duplication of classes for the students [which is equal to $\lambda _{i\ell }$ in Eq. (9)]: 12
  - Number of feedback loops for generation and evaluation of the timetables ${\varvec{X}}$: 240

- FS2014
  - Number of variables: 50,288
  - Number of constraints: 53,782
  - Number of classes not operated in the period for the makeup classes: 4
  - Computational time (for each optimization by a computer): 0.03–6,064 s
  - Duplication of classes for the students [which is equal to $\lambda _{i\ell }$ in Eq. (9)]: 10
  - Number of feedback loops for generation and evaluation of the timetables ${\varvec{X}}$: 382
- SS2014
  - Number of variables: 43,153
  - Number of constraints: 46,876
  - Number of classes not operated in the period for the makeup classes: 1
  - Computational time (for each optimization by a computer): 0.00–41,324 s
  - Duplication of classes for the students: 35
  - Number of feedback loops for generation and evaluation of the timetables ${\varvec{X}}$: 134

The results by the conventional scheduling techniques (i.e., hand working) are obtained as a comparison from the hearing survey to the staff, and they are also summarized as follows:

- The first semester of 2013 (we call FS2013)
  - $*$ Number of students: 994,
  - $*$ Total period of a makeup classes: 6 days
  - $*$ Number of classes requested by the lecturers: 257
  - Number of classes not operated in the period for the makeup classes: 4
  - Duplication of classes for the students: 44

**Figure 4:**
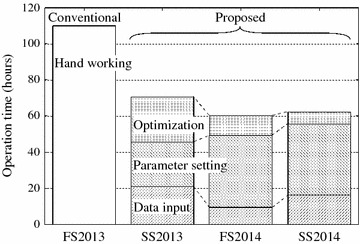
Results: operation time (h).

Figure 4 summarizes the operation time including both the operation by the staff and the computation by the computer. Here, the operation time is divided into the time for data input and for the parameter setting. In our all experiments, the modification of solution ${\varvec{X}}$ by the operators was never occurred. On the other hand, the priority parameter $b^\mathrm{S}_{ik}$, and $b^\mathrm{C}_{k}$ were updated by the operators with referring to ${\varvec{X}}'$ in the optimization loop.

### Discussion

We conducted hearing surveys for the staffs after the experiments. We asked open-ended questions on the workloads and the contents of operation. From the hearing surveys and the results described in "Results", the following points are observed:

- The physical load for the staff was reduced to about a half of FS2013.
- Timetables with a few duplication of classes were acquired in early iterations in the optimization loop.
- The model parameters such as $b^\mathrm{S}_{ik}$, $b^\mathrm{C}_{k}$ were modified by the operators in the optimization loop. On the other hand, the modification step of Figure 1 was not necessary for almost every iteration.
- Various timetables could be evaluated and compared by changing the model parameters.
- The computational time of the branch-and bound techniques can vary significantly depending on the model parameters corresponding to the implicit objective functions.
- The requests from the lectures are different among the three data sets, and it provided significant differences on the computation time of the branch-and-bound algorithm.
- The staffs could attend some other duties during the optimization process by a computer.
- Every acquired schedule was actually conducted in Toyama Prefectural University.

## Conclusions

In this paper, we focus on a timetabling problem of university makeup classes and construct a scheduling system based on man–machine interaction. Makeup classes which are requested by the lecturers have to be assigned to a specified time slot under the hard and soft constraints. A constraint based scheduling model is newly introduced and several parameters of the model are settled through the repetition of the solution evaluation by the operators. Through the numerical experiment with the actual data, workloads of the operators could be reduced to about a half of the case of the conventional hand working, and timetables with a few duplication of classes could be obtained. Every acquired timetables by the proposed method had a potential to be conducted in the actual use environment.

In our studies, the following issues have left to be investigated:

- Visualization of the factors which provides infeasible solutions in the constraints,
- Reduction of the computational costs by a computer, and
- Construct an expert model which can optimize the parameters of the meta model as an agent.
